# Supplementary material for: CRISPR-Cas9 engineering of the RAG2 locus via complete coding sequence replacement for therapeutic applications
Source: Nat Commun. 2023 Oct 27;14:6771. doi: 10.1038/s41467-023-42036-5 (PMC10611791; doi:10.1038/s41467-023-42036-5)
Supplement: Supplementary file 1 — Supplementary Information [file 41467_2023_42036_MOESM1_ESM.pdf]

Supplementary Information:

Supplementary Notes:

Supplementary Note 1 – Flow cytometry gating strategy and MFI calculation

Consistent with the methodology performed in the literature, the flow cytometry gates are determined by the level of episomal expression for each individual rAAV6 donor without the addition of the CRISPR-Cas9 RNP complex (termed rAAV6 only cells). This is done to exclude GFP^{low} cells that are only transiently expressing the GFP cassette in an episomal manner and are not true HDR^{+} (GFP^{high}) cells which are only detected upon the introduction of the CRISPR-Cas9 RNP complex (see Dever *et al.*[1], Bak *et al.*[2], and Bak *et al.*[3]). Lowering the gate uniformly to gate based on the untreated *Mock* samples would lead to the marked influx of HDR^{-} GFP^{low} cells into the calculation. Thus, we utilized a control sample that was treated only with the rAVV6 donor and no CRISPR-Cas9 RNP (RNP^{-}) complex for each rAVV6 donor individually. The calculated GFP^{+} values for these samples can be seen in Figs. 1A-B and 2A-B and Supplementary Figs. 2 and 4. GFP^{+} frequencies depicted in these figures are the values above that of their respective rAAV6 only cells.

When calculating the MFI, if the gate was lowered to include the entire GFP^{high} population or a uniform gate was drawn for all of the rAAV6 donors based on the Untreated sample, not only would a substantial number of cells that are actually HDR^{-} be included in the MFI calculation, but the number of cells that would now be included, based on the uniform gate, would also vary significantly between donors, thus skewing the calculated value (see Supplementary Fig. 1). This strategy would lower the calculated MFI across all donors, but the general trend of CDSR donors producing a higher MFI than the CSI donors would be broadly maintained. Thus, we determined

that the most logical and accurate way to determine the MFI was to gate each rAAV6 donor by its own RNP^- control and to calculate the MFI of only the GFP^{high} cells that are determined to be HDR^+ based on that sample's given control.

Supplementary Figures:

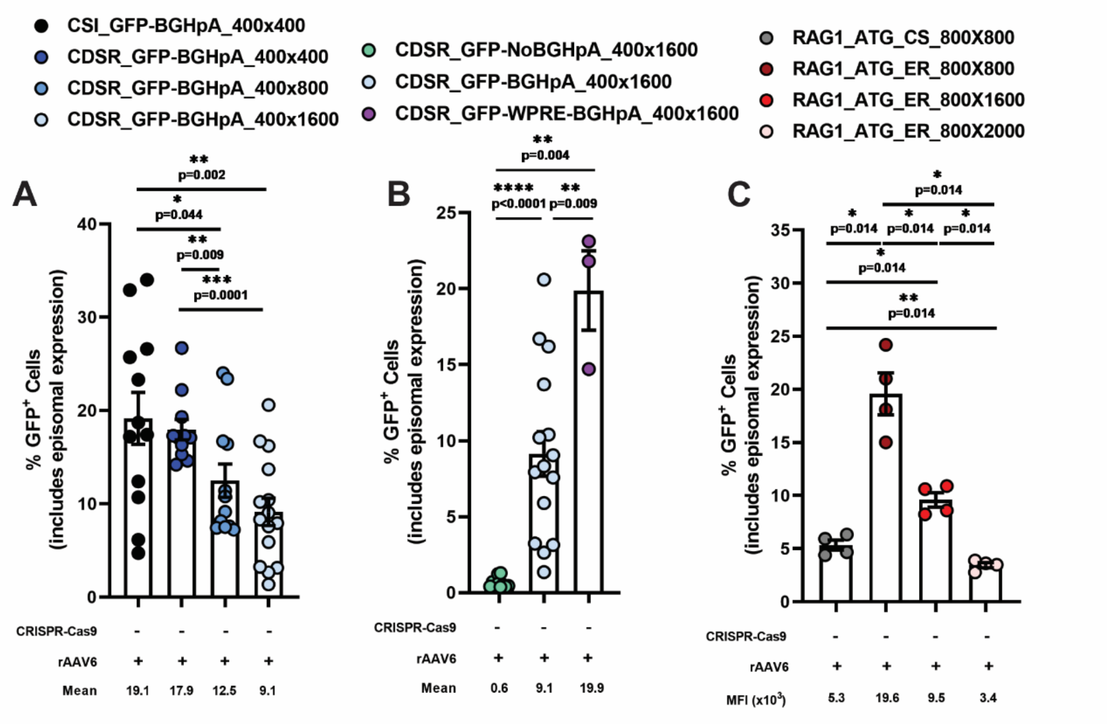

Supplementary Fig. 1. Variable episomal expression from different rAAV6 donors. These graphs depict the frequency of GFP$^+$ cells in rAAV6 only (RNP$^-$) samples when the gating was performed in a uniform manner based on the untreated *Mock* sample. Significant differences in GFP$^+$ frequencies of the rAAV6 only samples are observed, highlighting the fact that each rAAV6 donor produces a unique level of episomal expression. **(A)** *RAG2* donors depicted in Fig. 1; **(B)** *RAG2* donors depicted in Fig. 2; **(C)** *RAG1* donors depicted in Supplementary Fig. 3. Data are represented as mean ± SEM. * p<0.05, ** p<0.01, *** p<0.001, and **** p<0.0001 (Mann-Whitney one-sided test). Source data are provided as a Source Data file.

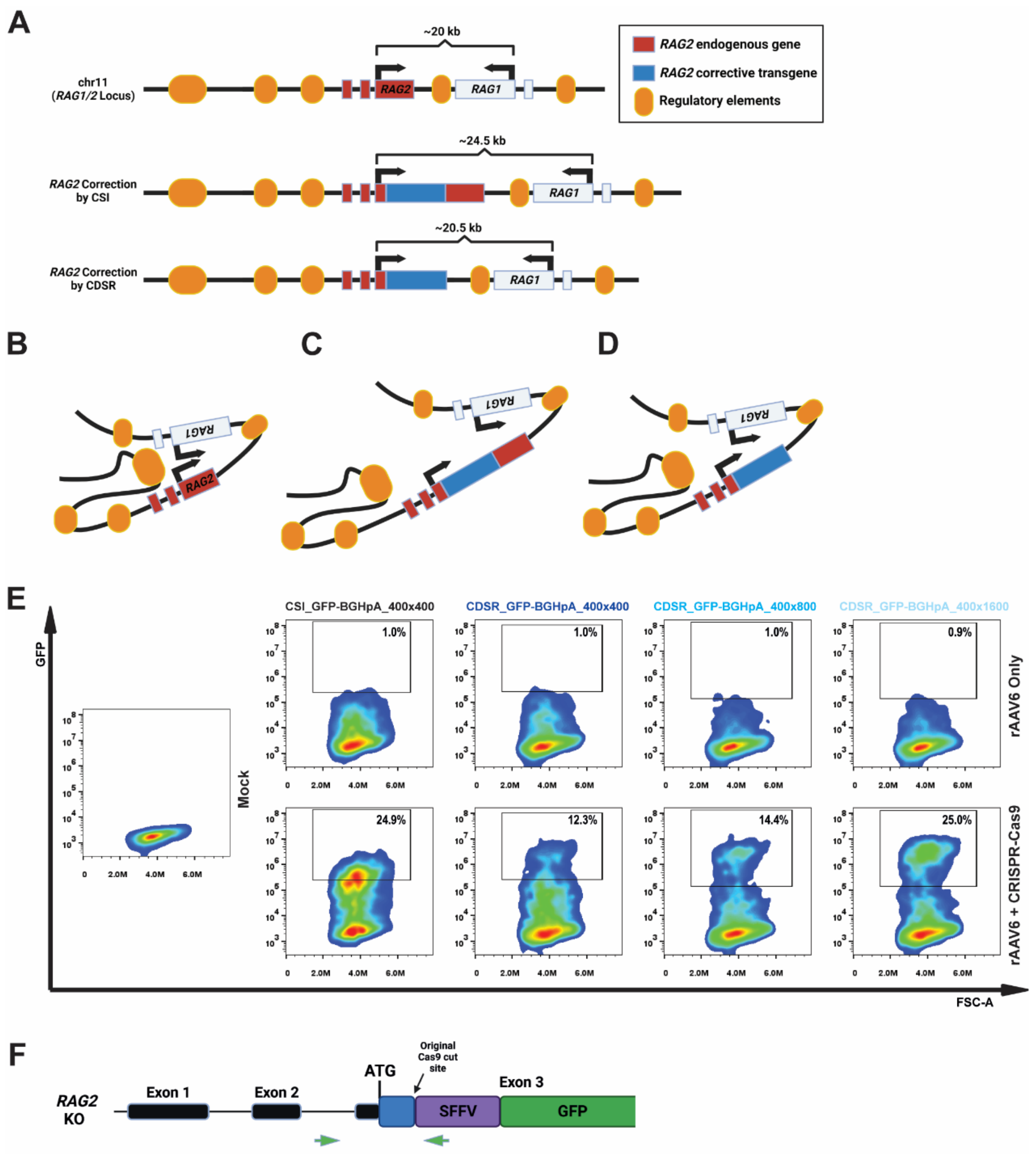

Supplementary Fig. 2. Different strategies for HDR at the *RAG2* locus: cut-site insertion vs. CDS replacement and adjusting homology arm length. (A) *Top row*: Schematic of the unedited *RAG* locus on chromosome 11. *Middle row*: Schematic of the *RAG* locus after cut-site insertion of the corrective *RAG2* transgene. This strategy leads to an insertion of ~4.8kb between the *RAG* genes. *Bottom row*: Schematic of the *RAG* locus after CDS replacement with the corrective *RAG2* transgene. This strategy limits the addition of added DNA to ~800bp between the *RAG* genes. **(B-D)** Representative schematics of potential super-enhancer structure depicting coordinate

expression of the *RAG* genes[7]. The schematics depict the looping of the regulatory elements to interact with both *RAG1* and *RAG2* promoter regions simultaneously. **(B)** Schematic depicting the super-enhancer structure of the unedited *RAG* locus. **(C)** Schematic depicting the super-enhancer structure of the *RAG* locus after cut-site insertion of the corrective *RAG2* transgene. **(D)** Schematic depicting the super-enhancer structure of the *RAG* locus after CDS replacement with the corrective *RAG2* transgene. Supplementary Figs. 2A-D were created with BioRender.com. **(E)** Representative flow cytometry plots of *RAG2* targeting with GFP *KO* donors in $CD34^+$ HSPCs two days post-CRISPR-Cas9/ rAAV6 editing. Each sample is gated based on its respective rAAV6 only sample. (*Top row*) cells treated only with the rAAV6 vectors; and (*Bottom row*) cells treated with both CRISPR-Cas9 complex and rAAV6. Gating determination is based on cells treated with the rAAV6 vector alone for each donor to determine the level of episomal expression. *Mock* plot is presented for comparative purposes and visualization of GFP^-, GFP^{low}, and GFP^{high} populations. **(F)** Schematic depicting the positioning of the ddPCR primer pair for detection of site-specific HDR of the KO GFP reporter gene cassette at the *RAG2* locus. Figure was created with BioRender.com.

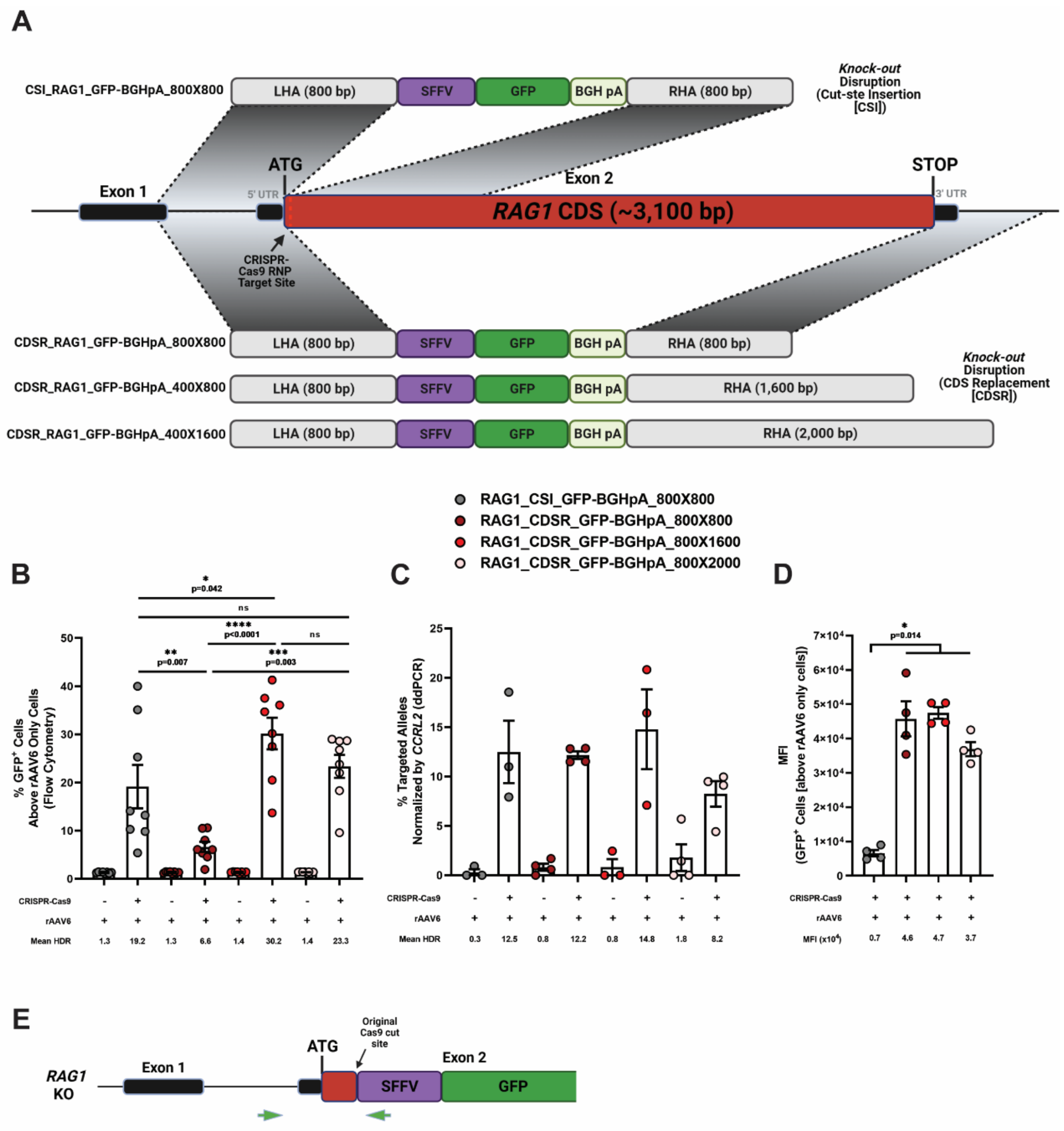

Supplementary Fig. 3. Different strategies for HDR at the *RAG1* locus: cut-site insertion vs. CDS replacement and adjusting homology arm length. (A) Schematic representation of *RAG1 KO* disruption donors containing a GFP reporter gene cassette under the control of an SFFV promoter and BGHpA sequence. Successful HDR of the RAG1_CSI_GFP-BGHpA_800x800 donor results in the integration of the reporter gene approximately 20bp downstream from the *RAG1* ATG start codon. Successful HDR of the three CDSR donors (RAG1_CDSR_GFP-BGHpA_800x800, RAG1_CDSR_GFP-BGHpA_800x1600, and RAG1_CDSR_GFP-BGHpA_800x2000) results in replacement of the entire endogenous *RAG1* CDS with

the reporter gene cassette (see Supplementary Table 2 for a more in-depth description of the donors). Figure was created with BioRender.com. **(B)** HDR frequencies analyzed by flow cytometry. See Supplementary Note 1 for a description of the gating strategy. RAG1_CSI_GFP-BGHpA_800x800 (N=8), RAG1_CDSR_GFP-BGHpA_800x800 (N=8), RAG1_CDSR_GFP-BGHpA_800x1600 (N=8), and RAG1_CDSR_GFP-BGHpA_800x2000 (N=8). Data are represented as mean ± SEM. * p<0.05, ** p<0.01, *** p<0.001, and **** p<0.0001 (Mann-Whitney one-sided test). **(C)** Site-specific HDR efficiencies at the *RAG1* locus measured by ddPCR and normalized by targeted *CCRL2* alleles. RAG1_CSI_GFP-BGHpA_800x800 ([rAAV6 only: N=3; CRISPR+AAV: N=3]), RAG1_CDSR_GFP-BGHpA_800x800 ([rAAV6 only: N=4; CRISPR+AAV: N=4]), RAG1_CDSR_GFP-BGHpA_800x1600 ([rAAV6 only: N=3; CRISPR+AAV: N=3]), and RAG1_CDSR_GFP-BGHpA_800x2000 ([rAAV6 only: N=4; CRISPR+AAV: N=4]). **(D)** MFI values of HDR$^+$ cells analyzed by flow cytometry. See Supplementary Note 1 for a description of the gating strategy. RAG1_CSI_GFP-BGHpA_800x800 (N=4), RAG1_CDSR_GFP-BGHpA_800x800 (N=4), RAG1_CDSR_GFP-BGHpA_800x1600 (N=4), and RAG1_CDSR_GFP-BGHpA_800x2000 (N=4). Data are represented as mean ± SEM. * p<0.05, ** p<0.01, *** p<0.001, and **** p<0.0001 (Mann-Whitney one-sided test). Source data are provided as a Source Data file. **(E)** Schematic depicting the positioning of the ddPCR primer pair for detection of site-specific HDR of the KO GFP reporter gene cassette at the *RAG1* locus. Figure was created with BioRender.com.

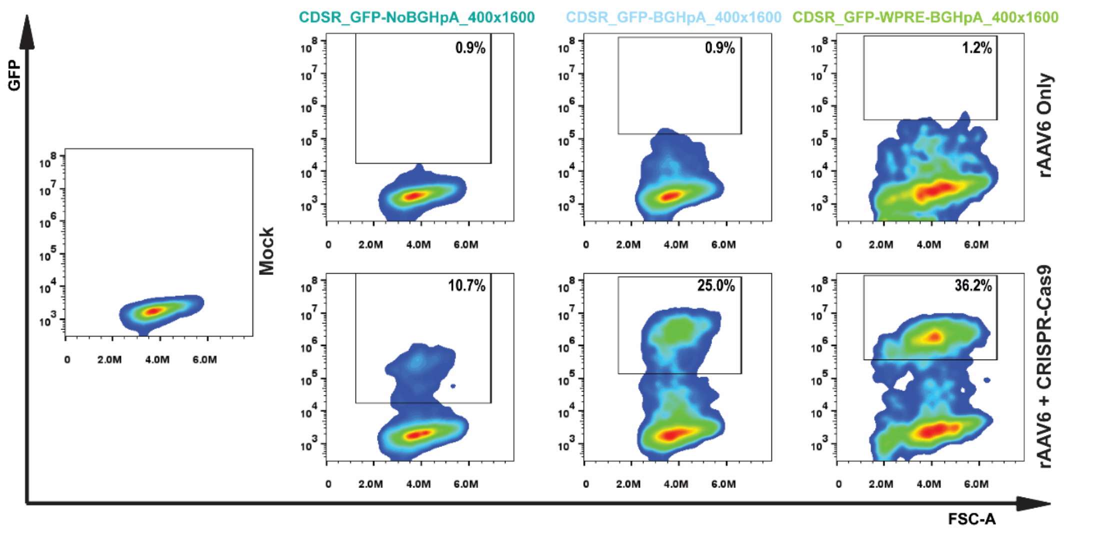

Supplementary Fig. 4. Effect of synthetic pA sequences and/or cis-acting PREs on transgene expression. Representative flow cytometry plots of *RAG2* targeting with GFP *KO* donors with different 3' UTRs in $CD34^+$ HSPCs two days post-CRISPR-Cas9/ rAAV6 editing. Each sample is gated based on its respective rAAV6 only sample. (*Top row*) cells treated only with the rAAV6 vectors; and (*Bottom row*) cells treated with both CRISPR-Cas9 complex and rAAV6. Gating determination is based on cells treated with the rAAV6 vector alone for each donor to determine the level of episomal expression. *Mock* plot is presented for comparative purposes and visualization of GFP^-, GFP^{low}, and GFP^{high} populations.

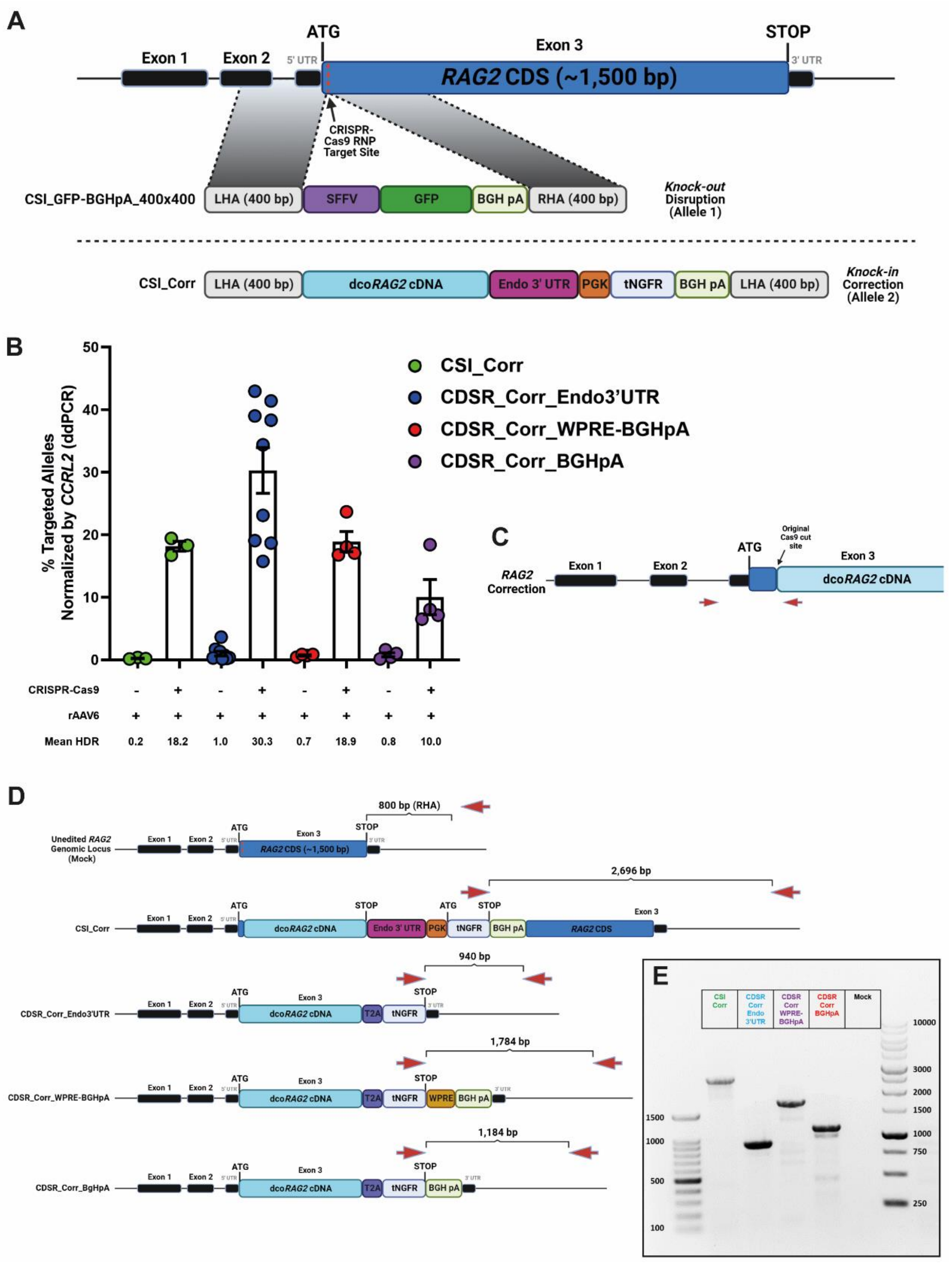

Supplementary Fig. 5. *KI* of *RAG2* Correction donor of in HD-derived CD34$^+$ HSPCs and biallelic targeting calibration. (A) Schematic representation of CSI *RAG2* rAAV6 donors for *KI-KO* biallelic correction simulation. (*Top to bottom*) CSI_GFP-BGHpA_400x400: *RAG2* disruption donor for gene *KO* containing a GFP reporter gene cassette under the control of a SFFV promoter and BGHpA sequence

(presented in Fig. 1A); and CSI_Corr: *RAG2* correction donor for *KI* of a dco*RAG2* cDNA sequence. Successful HDR of the two donors results in integration of the *KI* or *KO* donor approximately 43bp downstream from the *RAG2* ATG start codon (see Supplementary Table 1 for a more in-depth description of the donors). Figure was created with BioRender.com. **(B)** Site-specific HDR efficiencies at the *RAG2* locus measured by ddPCR and normalized by targeted *CCRL2* alleles. CSI_Corr (N=3), CDSR_Corr_Endo3'UTR (N=9), CDSR_Corr_BGHpA (N=4), and CDSR_Corr_WPRE-BGHpA (N=4). Data are represented as mean ± SEM. Source data are provided as a Source Data file. **(C)** Schematic depicting the positioning of the ddPCR primer pair for detection of site-specific HDR of the KI corrective *RAG2* donor at the *RAG2* locus. Figure was created with BioRender.com. **(D and E)** PCR amplification for locus-specific integration of the *RAG2* correction donors. **(D)** Schematic depicting the specific integration patterns of the different *KI* correction donors. Red arrows depict the positioning of the PCR primers. Figure was created with BioRender.com. **(E)** Gel image depicting the amplicon for the different *KI* correction donors. Amplicon sizes: CSI_Corr (2,696bp), CDSR_Corr_Endo3'UTR (940bp), CDSR_Corr_WPRE-BGHpA (1,784bp), and CDSR_Corr_BGHpA (1,184bp).

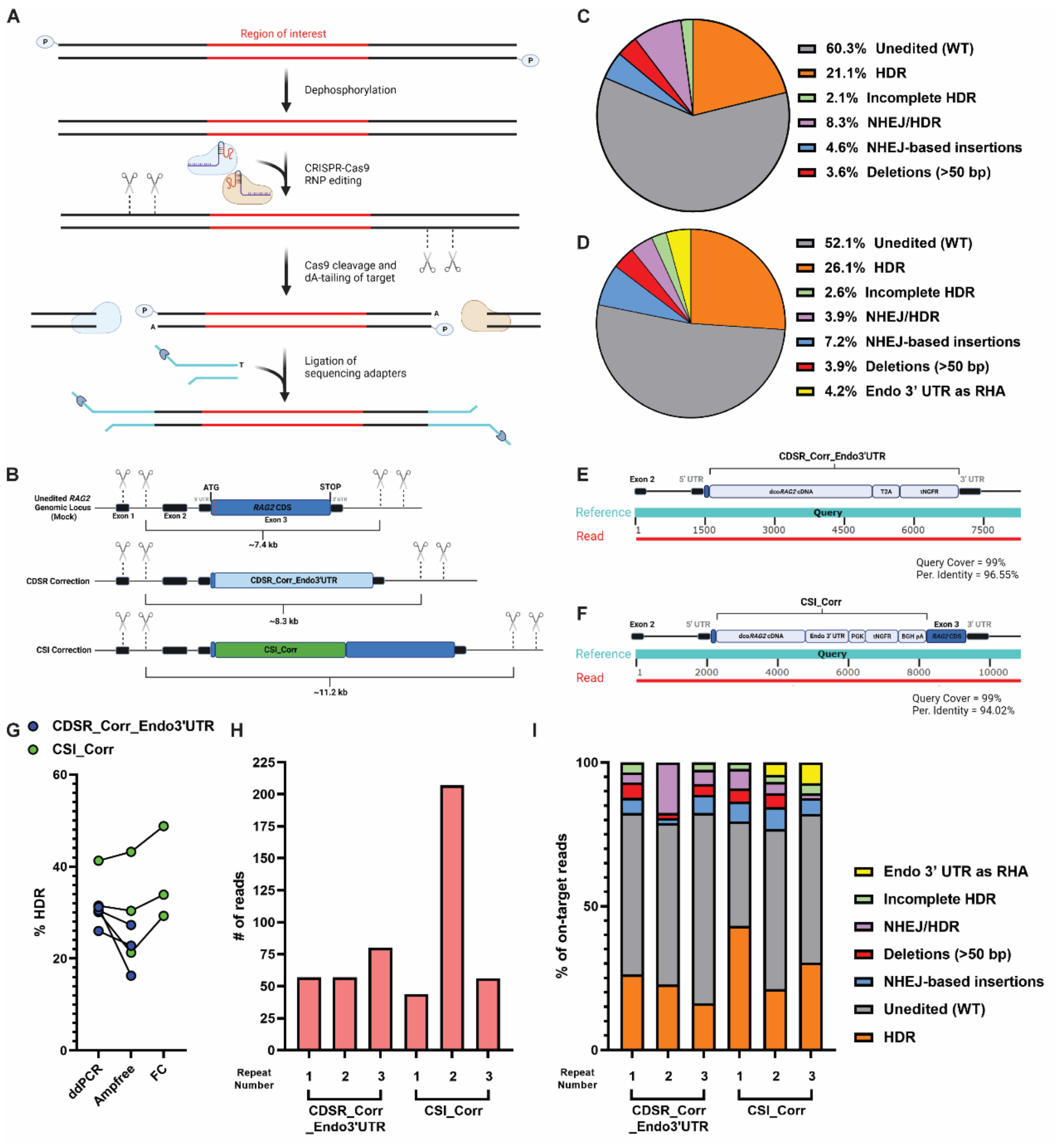

Supplementary Fig. 6. ITR-seq for specificity determination and amplification-free long-read ONT sequencing for classification of on-target genome-editing products. (A) Workflow of Cas9 digestion and library preparation for the *RAG2* locus. Two sgRNAs were used for each end of the locus of interest. **(B)** expected products and read lengths for the unedited locus (*top*), complete HDR after treatment with the CDSR_Corr_Endo3'UTR donor (*middle*), and complete HDR after treatment with the CSI_Corr donor (*bottom*). Supplementary Figs. 6A-B were created with BioRender.com. **(C-D)** Classification and average frequency of genome-editing

products identified by ONT sequencing of HSPCs edited with either CSI_Corr or CDSR_Corr_Endo3'UTR donors after Cas9 digestion (as depicted in *A* and *B*; possible gene-editing products depicted in Supplementary Fig. 1). **(C)** CDSR_Corr_Endo3'UTR genome-editing product average frequencies. N=3. **(D)** CSI_Corr genome-editing product average frequencies. N=3. **(E-F)** Depiction of representative alignment using BLAST tool for selected reads with perfect HDR of the CDSR_Corr_Endo3'UTR donor and CSI_Corr donor, *E* and *F*, respectively. Exact read sequences can be found in Supplementary Data 2. Supplementary Figs. 6E-F were created with BioRender.com. **(E)** Complete alignment of the selected read (red) to a reference sequence depicting perfect HDR with the CDSR_Corr_Endo3'UTR donor (teal) gave query coverage of 99% and percent identity of 96.55%. **(F)** Complete alignment of the selected read (red) to a reference sequence depicting perfect HDR with the CSI_Corr donor (teal) gave query coverage of 99% and a percent identity of 94.02%. **(G)** Comparison of HDR frequencies for CSI_Corr determined by FC, ddPCR, and amplification-free (Ampfree) long-read ONT sequencing (N=3) and for CDSR_Corr_Endo3'UTR determined by ddPCR and amplification-free long-read ONT sequencing (N=3). **(H)** Raw number of on-target reads attained from amplification-free long-read ONT sequencing for each individual repeat. **(I)** The frequency distribution of on-target genome-editing products identified by Ampfree long-read ONT sequencing for each individual repeat. Source data are provided as a Source Data file.

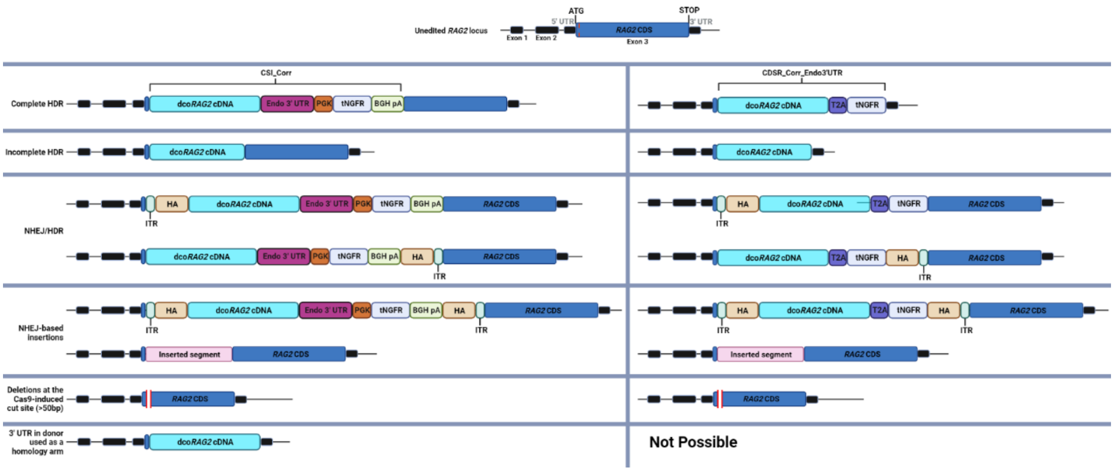

Supplementary Fig. 7. ONT long-read sequencing gene-editing product possibilities. Representative diagram depicting the different detected gene-editing products after ONT long-read sequencing for cells edited with the CSI_Corr (left) and CDSR_Corr_Endo3'UTR (right) donors. Complete HDR: HDR that occurs as expected on both ends of the donor. Incomplete HDR: HDR that occurs as expected on one end of the donor but only incorporates part of the donor. NHEJ/HDR: Integration that occurs via HDR on one end of the donor and via NHEJ on the other end. This was observed in both orientations as depicted above. Additionally, on the NHEJ end, the donor can be incorporated multiple times in a row (not shown above). NHEJ-based insertions: Integration that occurs via NHEJ on both ends of the donor or cases where large DNA segments (>50bp) were incorporated into the Cas9-induced cut site via NHEJ. Deletions (>50bp): Reads where no integration was detected yet there were more than 50bp deleted from the Cas9-induced cut site. 3' UTR as a homology arm: This scenario is only possible with the CSI_Corr donor with reads detected, where the endogenous 3' UTR sequence inside the donor acts as an RHA leading to early cessation of HDR and does not lead to integration of the entire donor. Figure was created with BioRender.com.

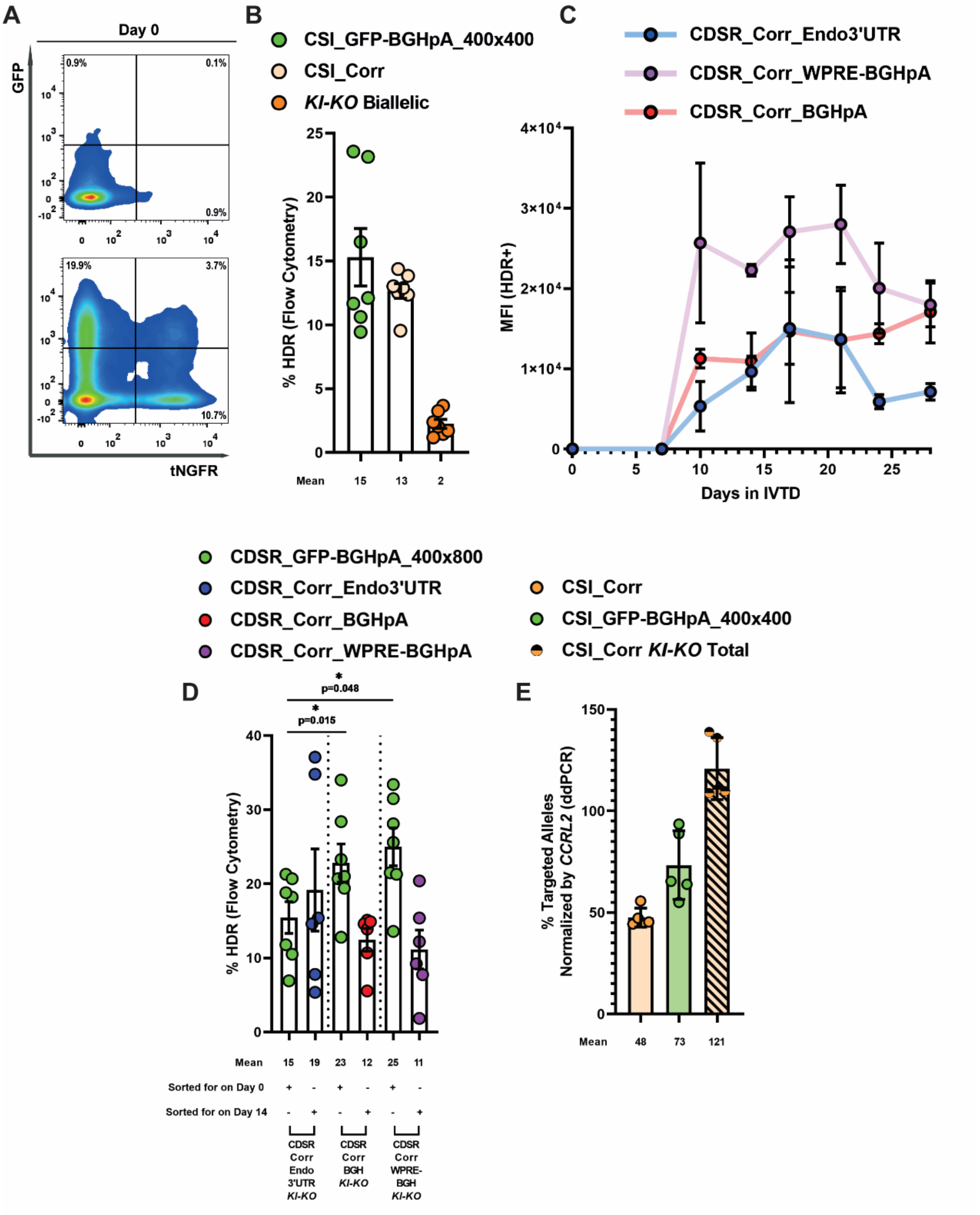

Supplementary Fig. 8. *KI-KO* Simulation of Functional Gene Correction of *RAG2* in HD-derived CD34⁺ HSPCs Using Two-part Enrichment Strategy. (A) FACS

enrichment approach on day 0 for *KI-KO* multiplexed HDR gene-targeted $CD34^+$ HSPCs post-CRISPR-Cas9/rAAV6 editing with a combination of either CSI_GFP-BGHpA_400x400 and CSI_Corr. Representative FACS plots of: (*Top*) cells transduced with only the two rAAV6 donors (no CRISPR-Cas9 complex) and (*Bottom*) cells treated with the CRISPR-Cas9 and two rAAV6 donors together. Cells were enriched for double-positive $tNGFR^+/GFP^+$ expression indicative of biallelic integration of the two distinct DNA donors. Gating determination is based on cells treated with the rAAV6 vector alone for each donor to determine the level of episomal expression. **(B)** HDR efficiencies as measured by flow cytometry for the *KI* and *KO* donors as well as the double-positive biallelic frequencies ($tNGFR^+/GFP^+$) in the multiplexed *KI-KO* HDR treatment with CSI_ GFP-BGHpA_400x400 and CSI_Corr donors on day 0. **(C)** Plot depicting the transgene expression patterns for CDSR_Corr_Endo3'UTR, CDSR_Corr_BGHpA, and CDSR_Corr_WPRE-BGHpA donors over 28 days of IVTD. (N=4). Data are represented as mean ± SEM. **(D)** Individual HDR efficiencies as measured by flow cytometry for the *KI* and *KO* donors in the multiplexed *KI-KO* HDR treatment with CDSR_GFP-BGHpA_400x800 (on day 0) together with CDSR_Corr_Endo3'UTR, CDSR_Corr_BGHpA, or CDSR_Corr_WPRE-BGHpA donors (on day 14). **(E)** Site-specific multiplex HDR efficiencies at the *RAG2* locus measured by ddPCR and normalized by targeted *CCRL2* alleles. After extraction of genomic DNA, quantification of targeted alleles with *KI* and *KO* donors were conducted individually and the sum of the two indicated 100% enrichment of *KI-KO* cells. (N=5). Data are represented as mean ± SEM. Source data are provided as a Source Data file.

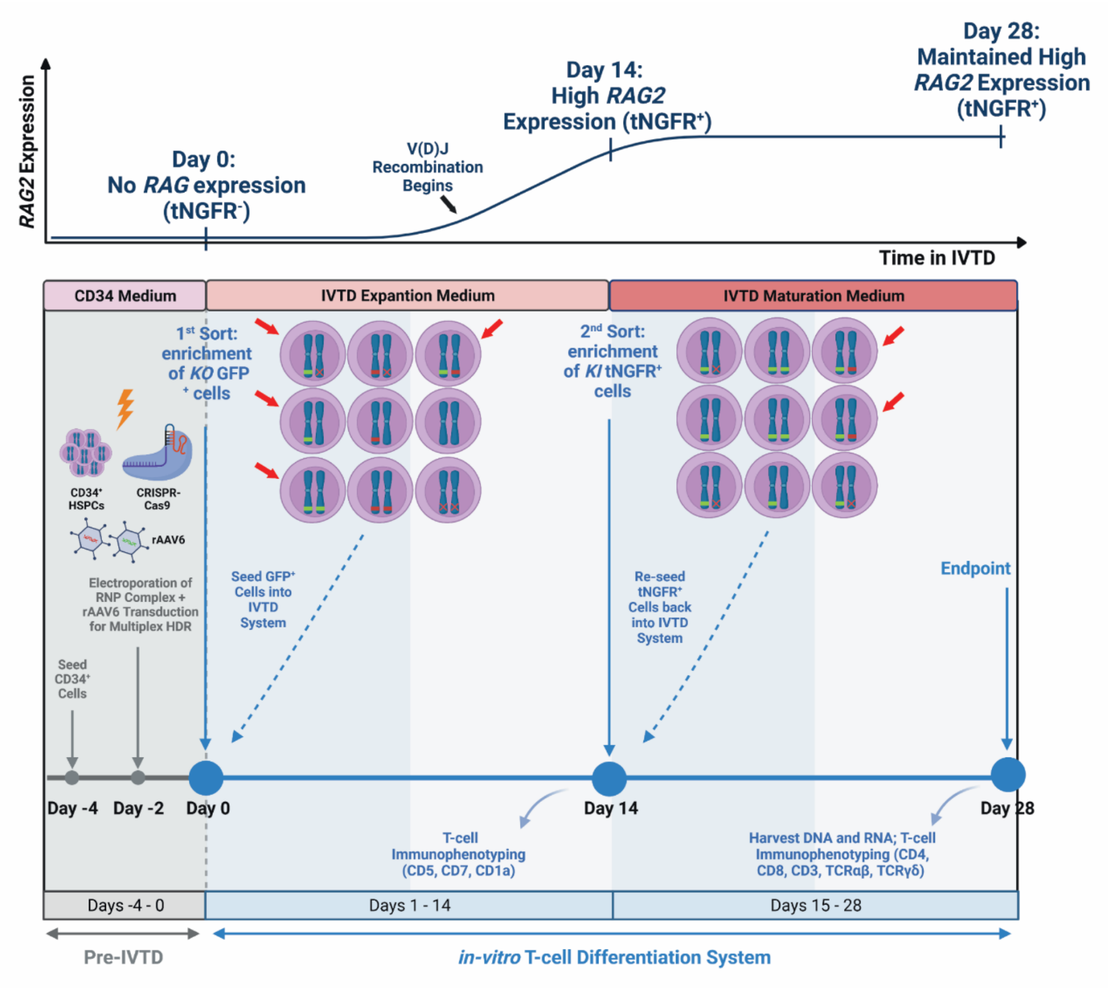

Supplementary Fig. 9. *KI-KO* two-step enrichment strategy. Schematic depicting the two-step enrichment strategy of CDSR *KI-KO* cells. The CSI_Corr donor is sorted for biallelic *KI-KO* double-positive tNGFR$^+$/GFP$^+$ expression on day 0 (two days post-editing), whereas all three CDSR correction groups express GFP on day 0, however, do not yet express tNGFR since the *RAG2* locus does not undergo transcription until later in the T-cell differentiation process. Therefore, for the CDSR donors, enrichment of GFP$^+$ cells is conducted, and cells are seeded into the IVTD system. On day 14 of IVTD, all three CDSR correction groups express tNGFR at different levels. Enrichment of the CDSR correction groups for tNGFR$^+$ cells is conducted, producing a homogenous double-positive tNGFR$^+$/GFP$^+$ population indicative of *KI-KO* biallelic integration. In addition, all groups, including the *Mock* samples are sorted for CD7$^+$

expression. The cells are then seeded back into the IVTD system for another 14 days. Immunophenotyping of the T-cell developmental progression via flow cytometry was performed on days 14 and 28 of IVTD of Mock and *KI-KO* populations. Cells were stained for CD7, CD5, and CD1a expression on day 14 of IVTD and for CD4, CD8, CD3, and TCRγδ expression on day 28 of IVTD. Additionally, cell populations were sampled for DNA and RNA on day 28 for ddPCR and qRT-PCR analyses. Figure was created with BioRender.com.

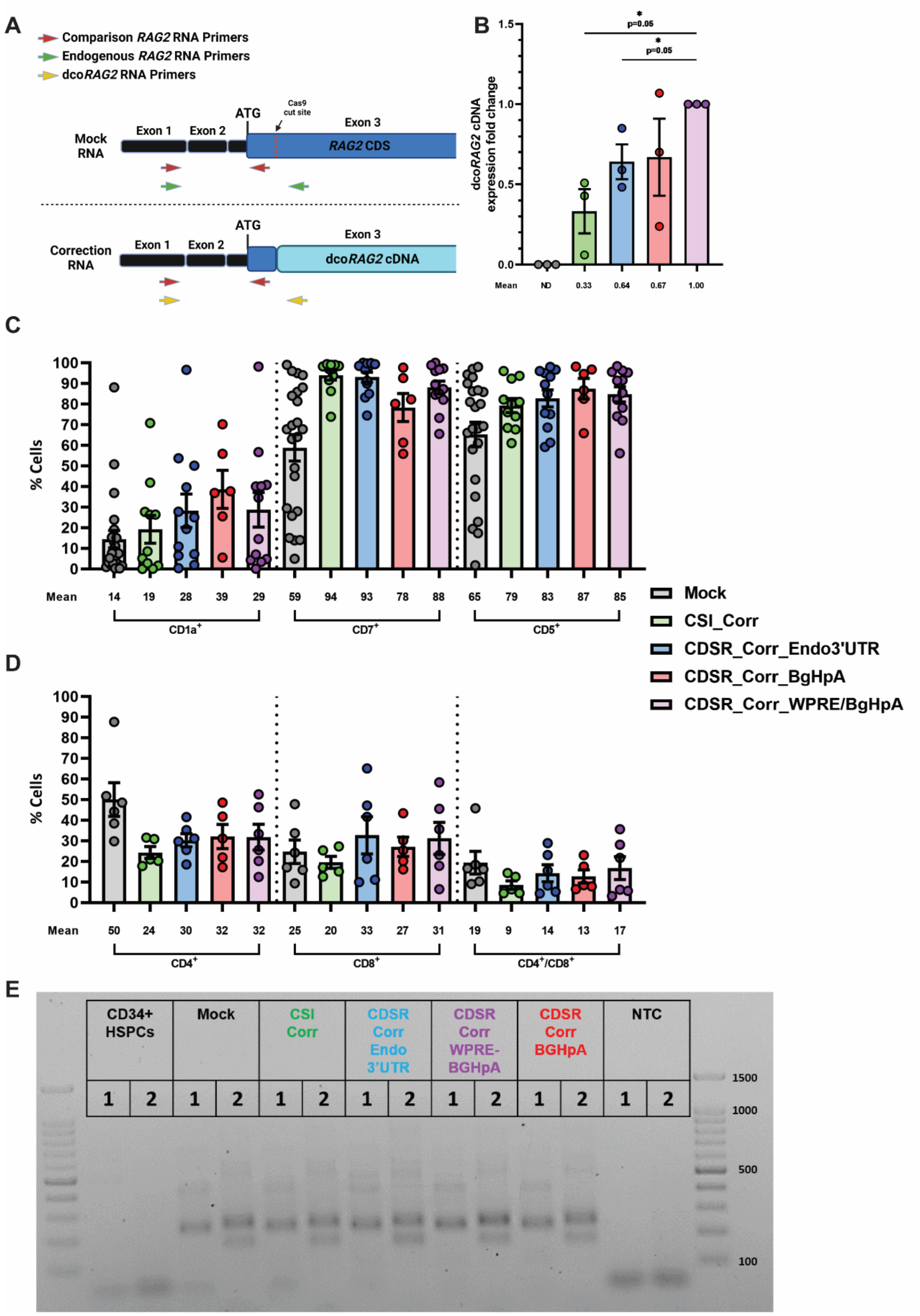

Supplementary Fig. 10. *KI-KO* correction simulation cells develop into CD3^{+}TCRγδ^{+} and CD3^{+}TCRαβ^{+} T cells in the IVTD system. (A) Schematic depicting the positioning of the qRT-PCR primer pairs. The red primers are used for

quantification of total *RAG2* expression (comparison between dco*RAG2* and endogenous *RAG2* [depicted in Fig. 4B]). The green primers are used for quantification of endogenous *RAG2* expression (depicted in Fig. 4A). The yellow primers are used for the quantification of dco*RAG2* expression (depicted in Supplementary Fig. 10B). Figure was created with BioRender.com. **(B)** qRT-PCR quantification of dco*RAG2* cDNA expression in the *RAG2 KI-KO* cells compared to *Mock* cells on day 28 of IVTD (using yellow primer pair depicted in Supplementary Fig. 10A). Expression fold change is plotted relative to CDSR_Corr_WPRE-BGHpA and samples with no expression detected are plotted as ND. (N=3). Data are represented as mean ± SEM. * p<0.05, ** p<0.01, *** p<0.001, and **** p<0.0001 (Mann-Whitney one-sided test). **(C and D)** Summary of immunophenotyping during IVTD for *Mock* and *RAG2 KI-KO* correction populations. Gating strategy depicted in Supplementary Fig. 11. **(C)** T-cell marker frequencies of CD5, CD7, and CD1a populations on day 14. *Mock* (N=23), CSI_Corr (N=11), CDSR_Corr_Endo3'UTR (N=12), CDSR_Corr_BGHpA (N=6), and CDSR_Corr_WPRE-BGHpA (N=12). **(D)** T-cell marker frequencies of CD4, CD8, and $CD4^+CD8^+$ populations on day 28 of IVTD. *Mock* (N=6), CSI_Corr (N=5), CDSR_Corr_Endo3'UTR (N=6), CDSR_Corr_BGHpA (N=5), and CDSR_Corr_WPRE-BGHpA (N=6). Data are represented as mean ± SEM. **(E)** PCR amplification of TRG V(D)J recombination using primers that flank the V-J genomic regions for: (*left to right*) $CD34^+CD3^-$ HD-derived HSPCs, and *Mock*, CSI_Corr, CDSR_Corr_Endo3'UTR, CDSR_Corr_WPRE-BGHpA, and CDSR_Corr_BGHpA *KI-KO* cells on day 28 of IVTD. Lane numbers 1 and 2 represent different pools of Vγ and Jγ primer combinations (primer sequences found in Table 2). 1 represents $V\gamma_{\_9\_2}$ and $V\gamma_{\_11}$ primers together in one tube with the three Jγ primers and 2 represents the $V\gamma_{\_f1}$ and $V\gamma_{\_10\text{-}2}$ primers together in one tube with the three Jγ primers. Primers are all

10 µM and pooled together by adding equal volumes of each required primer. Source data are provided as a Source Data file.

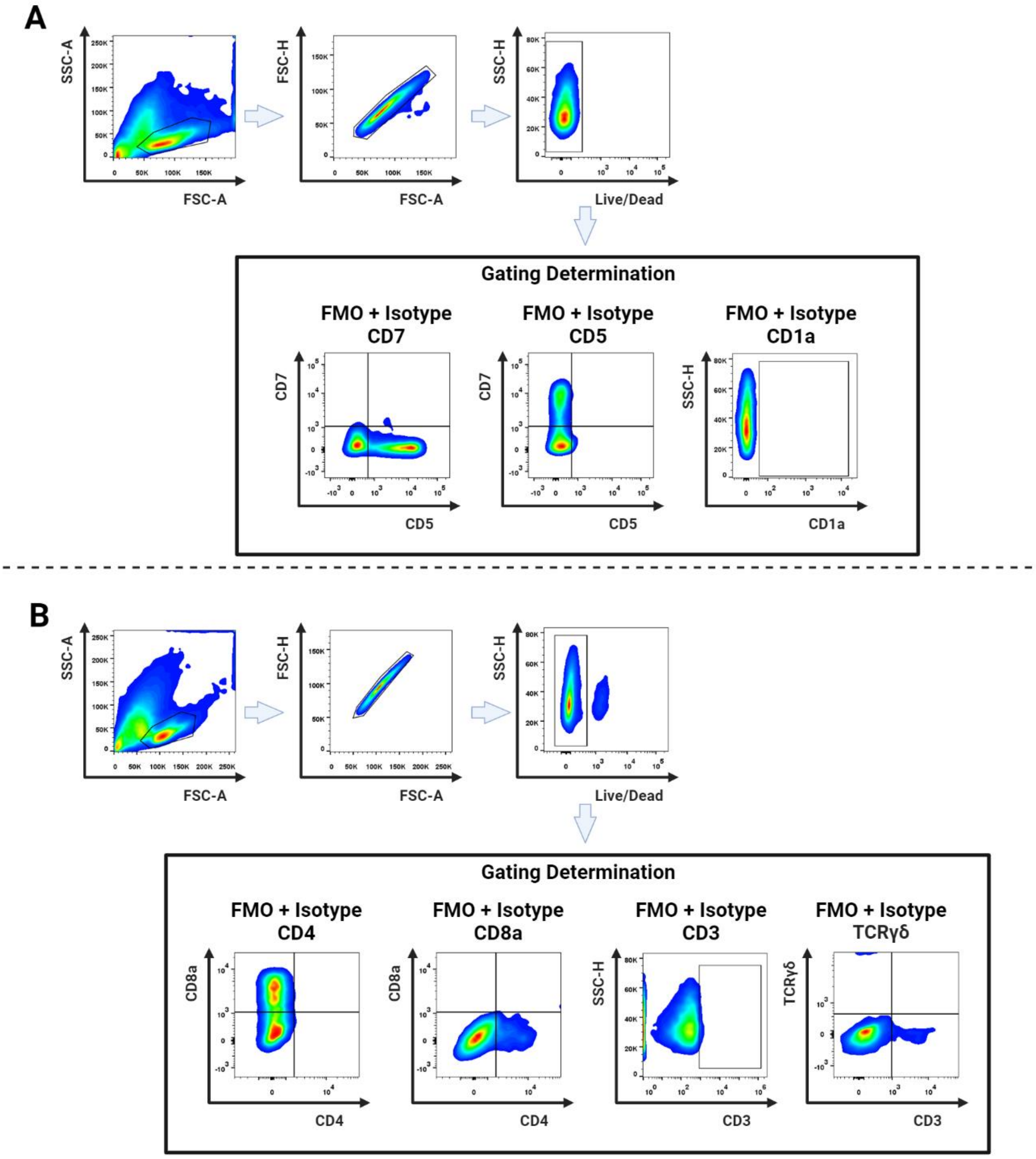

Supplementary Fig. 11. T-cell immunophenotyping gating strategy. Immunophenotyping via flow cytometry was conducted on cells that were incubated in the IVTD system on days 14 and 28. Viability staining was performed on all collected

cells at both time points. Gating strategies were based on fluorescence minus one (FMO) plus isotype control (at equivalent concentration to its antibody pair) samples. **(A)** On day 14 of IVTD, cells were analyzed for their expression of CD7, CD5, and CD1a. **(B)** On day 28 of IVTD, cells were cells were analyzed for their expression of CD4, CD8a, CD3, and TCRγδ.

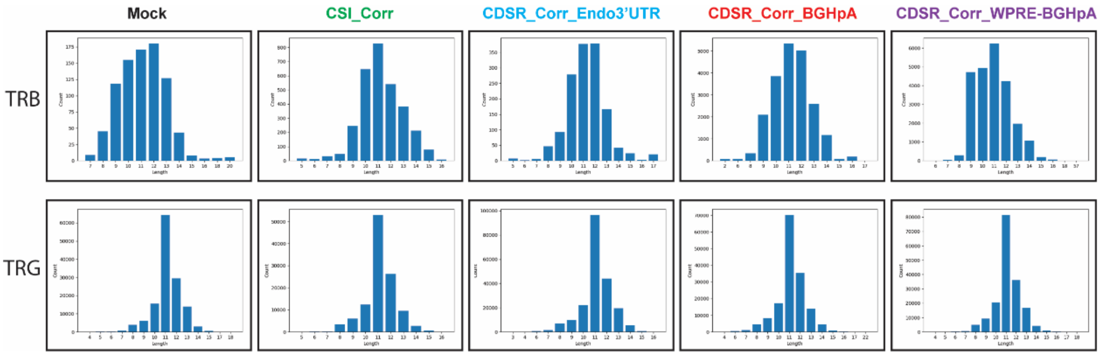

Supplementary Fig. 12. Expression of dco*RAG2* cDNA induces normal TRB and TRG repertoire development. Representative histograms of CDR3 length distributions from unique TRB and TRG sequences expressed in *Mock* and *RAG2 KI-KO* correction populations on days 28 of IVTD.

Supplementary Tables:

Donor Name	Description	Schematic
CSI_GFP-BGHpA_400X400	Donor for *RAG2* gene *KO*. Cut site integration of GFP reporter gene cassette under the control of an SSFV promoter followed by a BGHpA sequence with 400 bp LHA and RHA.	LHA (400 bp) – SFFV – GFP – BGH pA – RHA (400 bp)
CDSR_GFP-BGHpA_400X400	Donor for *RAG2* gene *KO*. CDS replacement integration of GFP reporter gene cassette under the control of an SSFV promoter followed by a BGHpA sequence with 400 bp LHA and RHA.	LHA (400 bp) – SFFV – GFP – BGH pA – RHA (400 bp)
CDSR_GFP-BGHpA_400X800	Donor for *RAG2* gene *KO*. CDS replacement integration of GFP reporter gene cassette under the control of an SSFV promoter followed by a BGHpA sequence with 400 bp LHA and 800 bp RHA.	LHA (400 bp) – SFFV – GFP – BGH pA – RHA (800 bp)
CDSR_GFP-BGHpA_400X1600	Donor for *RAG2* gene *KO*. CDS replacement integration of GFP reporter gene cassette under the control of an SSFV promoter followed by a BGHpA sequence with 400 bp LHA and 1,600 bp RHA.	LHA (400 bp) – SFFV – GFP – BGH pA – RHA (1,600 bp)
CDSR_GFP-NoBGHpA_400X1600	Donor for *RAG2* gene *KO*. CDS replacement integration of GFP reporter gene cassette under the control of an SSFV promoter without a BGHpA sequence with 400 bp LHA and 1,600 bp RHA.	LHA (400 bp) – SFFV – GFP – RHA (1,600 bp)
CDSR_GFP-WPRE-BGHpA_400X1600	Donor for *RAG2* gene *KO*. CDS replacement integration of GFP reporter gene cassette under the control of an SSFV promoter followed by WPRE and BGHpA sequences with 400 bp LHA and 1,600 bp RHA.	LHA (400 bp) – SFFV – GFP – WPRE – BGH pA – RHA (1,600 bp)
CSI_Corr	Donor for *RAG2* CDS correction. Cut site integration of dco*RAG2* cDNA under the control of the endogenous *RAG2* promoter followed by a tNGFR reporter gene cassette under the control of a PGK promoter followed by a BGHpA sequence with 400 bp LHA and RHA.	LHA (400 bp) – dco*RAG2* cDNA – Endo 3' UTR – PGK – tNGFR – BGH pA – LHA (400 bp)
CDSR_Corr_Endo3'UTR	Donor for *RAG2* CDS correction. Gene replacement integration of dco*RAG2* cDNA under the control of the endogenous *RAG2* promoter and 3' UTR. The stop codon of the dco*RAG2* cDNA is abolished leading to in-frame transcription of the T2A self-cleaving peptide sequence and the tNGFR reporter gene cassette. HDR integration is mediated by with a 400 bp LHA and 800 bp RHA.	LHA (400 bp) – dco*RAG2* cDNA – T2A – tNGFR – RHA (800 bp)
CDSR_Corr_BGHpA	Donor for *RAG2* CDS correction. Gene replacement integration of dco*RAG2* cDNA under the control of the endogenous *RAG2* promoter. The stop codon of the dco*RAG2* cDNA is abolished leading to in-frame transcription of the T2A self-cleaving peptide sequence and the tNGFR reporter gene cassette followed by a BGHpA sequence. HDR integration is mediated by with a 400 bp LHA and 800 bp RHA.	LHA (400 bp) – dco*RAG2* cDNA – T2A – tNGFR – BGH pA – RHA (800 bp)
CDSR_Corr_WPRE-BGHpA	Donor for *RAG2* CDS correction. Gene replacement integration of dco*RAG2* cDNA under the control of the endogenous *RAG2* promoter. The stop codon of the dco*RAG2* cDNA is abolished leading to in-frame transcription of the T2A self-cleaving peptide sequence and the tNGFR reporter gene cassette followed by WPRE and BGHpA sequences. HDR integration is mediated by with a 400 bp LHA and 800 bp RHA.	LHA (400 bp) – dco*RAG2* cDNA – T2A – tNGFR – WPRE – BGH pA – RHA (800 bp)

Supplementary Table 1. *RAG2* rAAV6 donor descriptions. Table was created with BioRender.com.

Donor Name	Description	Schematic
RAG1_CSI_GFP-BGHpA_800X800	Donor for *RAG1* gene *KO*. Cut site integration of GFP reporter gene cassette under the control of an SSFV promoter followed by a BGHpA sequence with 800 bp LHA and RHA.	LHA (800 bp) – SFFV – GFP – BGH pA – RHA (800 bp)
RAG1_CDSR_GFP-BGHpA_800X800	Donor for *RAG1* gene *KO*. CDS replacement integration of GFP reporter gene cassette under the control of an SSFV promoter followed by a BGHpA sequence with 800 bp LHA and RHA.	LHA (800 bp) – SFFV – GFP – BGH pA – RHA (800 bp)
RAG1_CDSR_GFP-BGHpA_800X1600	Donor for *RAG1* gene *KO*. CDS replacement integration of GFP reporter gene cassette under the control of an SSFV promoter followed by a BGHpA sequence with 800 bp LHA and 1,600 bp RHA.	RHA (800 bp) – SFFV – GFP – BGH pA – RHA (1,600 bp)
RAG1_CDSR_GFP-BGHpA_800X2000	Donor for *RAG1* gene *KO*. CDS replacement integration of GFP reporter gene cassette under the control of an SSFV promoter followed by a BGHpA sequence with 800 bp LHA and 2,000 bp RHA.	RHA (800 bp) – SFFV – GFP – BGH pA – RHA (2,000 bp)

Supplementary Table 2. *RAG1* rAAV6 donor descriptions. Table was created with BioRender.com.

**P5 Adapters**	
**Adapter I.D.**	**Sequence 5'→3'**
A01 GS	AATGATACGGCGACCACCGAGATCTACACTAGATCGC(N:25252525)(N)W(N)(N)W(N)(N)ACACTCTTTCCCTACACGACGCTCTTCCGATC*T
A02 GS	AATGATACGGCGACCACCGAGATCTACACCTCTCTATNNWNNWNNACACTCTTTCCCTACACGACGCTCTTCCGATC*T
A03 GS	AATGATACGGCGACCACCGAGATCTACACTATCCTCTNNWNNWNNACACTCTTTCCCTACACGACGCTCTTCCGATC*T
A04 GS	AATGATACGGCGACCACCGAGATCTACACAGAGTAGANNWNNWNNACACTCTTTCCCTACACGACGCTCTTCCGATC*T
Common Adapter GS	/5Phos/GATCGGAAGAGC*C*A
**ITR-specific Primer**	
**Primer I.D.**	**Sequence 5'→3'**
GSP_ITR3.AAV2	TGACTGGAGTCCTCTCTATGGGCAGTCGGTGATACAAGGAACCCCTAGTGATGGAGTTGGCC
**P5 Primers**	
**Primer I.D.**	**Sequence 5'→3'**
A01-P5-Fwd	AATGATACGGCGACCACCGAGATCTACACTAGATCGC
A02-P5-Fwd	AATGATACGGCGACCACCGAGATCTACACCTCTCTAT
A03-P5-Fwd	AATGATACGGCGACCACCGAGATCTACACTATCCTCT
A04-P5-Fwd	AATGATACGGCGACCACCGAGATCTACACAGAGTAGA
**P7 Primers**	
**Primer I.D.**	**Sequence 5'→3'**
P701 GS	CAAGCAGAAGACGGCATACGAGATTCGCCTTAGTGACTGGAGTCCTCTCTATGGGCAGTCGGTGA
P702 GS	CAAGCAGAAGACGGCATACGAGATCTAGTACGGTGACTGGAGTCCTCTCTATGGGCAGTCGGTGA
P703 GS	CAAGCAGAAGACGGCATACGAGATTTCTGCCTGTGACTGGAGTCCTCTCTATGGGCAGTCGGTGA

P704 GS	CAAGCAGAAGACGGCATACGAGATGCTCAGGAGTGACTGGA GTCCTCTCTATGGGCAGTCGGTGA
P705 GS	CAAGCAGAAGACGGCATACGAGATAGGAGTCCGTGACTGGA GTCCTCTCTATGGGCAGTCGGTGA
P706 GS	CAAGCAGAAGACGGCATACGAGATCATGCCTAGTGACTGGAG TCCTCTCTATGGGCAGTCGGTGA
P707 GS	CAAGCAGAAGACGGCATACGAGATGTAGAGAGGTGACTGGA GTCCTCTCTATGGGCAGTCGGTGA
P708 GS	CAAGCAGAAGACGGCATACGAGATCCTCTCTGGTGACTGGAG TCCTCTCTATGGGCAGTCGGTGA
**Custom Primers for NGS**	
**Primer I.D.**	**Sequence 5'→3'**
Index1	ATCACCGACTGCCCATAGAGAGGACTCCAGTCAC
Read2	GTGACTGGAGTCCTCTCTATGGGCAGTCGGTGAT

Supplementary **Table 3. ITR-seq adapters and primers**

				**# of reads**			
	**Start**	**End**		**CSI_Corr**	**CDSR_Corr_ Endo3'UTR**	***Mock***	
**Chr11**	**36593721**	**36594369**	***RAG2* CDS**	**372**	**2023**	**3**	**On-target**
Chr3	182671736	182671811	Intergenic	60	0	0	Off-target
Chr7	48361166	48361263	Intronic	0	63	1	Off-target

Supplementary **Table 4. ITR-seq detection of NHEJ-based insertions throughout the genome**

**sgRNA name**	**Location**	**Sequence 5'→3'**
RAG268P	Downstream to *RAG2*	ATATACCTTGGGCTGAGCTG
RAG224P	Downstream to *RAG2*	CCATTAGTCTTCCTCCCATC
RAG224N	Upstream to *RAG2*	TTGAACCATGTTACAAGAGG
RAG222N	Upstream to *RAG2*	TTAGCGGCAAAGATTCAGAG

Supplementary **Table 5. sgRNA sequences for Cas9 Digestion**

References

1. Dever DP, Bak RO, Reinisch A, et al. CRISPR/Cas9 β-globin gene targeting in human haematopoietic stem cells. *Nature*. 2016;539(7629):384–389.

2. Bak RO, Dever DP, Porteus MH. CRISPR/Cas9 genome editing in human hematopoietic stem cells. *Nat Protoc*. 2018;13(2):358–376.

3. Bak RO, Dever DP, Reinisch A, et al. Multiplexed genetic engineering of human hematopoietic stem and progenitor cells using CRISPR/Cas9 and AAV6. *Elife*. 2017;6:.

4. Breton C, Clark PM, Wang L, Greig JA, Wilson JM. ITR-Seq, a next-generation sequencing assay, identifies genome-wide DNA editing sites in vivo following adeno-associated viral vector-mediated genome editing. *BMC Genomics*. 2020;21(1):239.

5. Tsai SQ, Nguyen NT, Malagon-Lopez J, et al. CIRCLE-seq: a highly sensitive in vitro screen for genome-wide CRISPR-Cas9 nuclease off-targets. *Nat Methods*. 2017;14(6):607–614.

6. Li H. Minimap2: pairwise alignment for nucleotide sequences. *Bioinformatics*. 2018;34(18):3094–3100.

7. Yu W, Misulovin Z, Suh H, et al. Coordinate regulation of RAG1 and RAG2 by cell type-specific DNA elements 5’ of RAG2. *Science*. 1999;285(5430):1080–1084.
