# Supplementary material for: CRISPR-Cas9 engineering of the RAG2 locus via complete coding sequence replacement for therapeutic applications
Source: Nat Commun. 2023 Oct 27;14:6771. doi: 10.1038/s41467-023-42036-5 (PMC10611791; doi:10.1038/s41467-023-42036-5)
Supplement: Supplementary file 2 — Reporting Summary [file 41467_2023_42036_MOESM2_ESM.pdf]

nature portfolio

Corresponding author(s): Ayal Hendel

Last updated by author(s): Sep 27, 2023

Reporting Summary

Nature Portfolio wishes to improve the reproducibility of the work that we publish. This form provides structure for consistency and transparency in reporting. For further information on Nature Portfolio policies, see our Editorial Policies and the Editorial Policy Checklist.

Statistics

For all statistical analyses, confirm that the following items are present in the figure legend, table legend, main text, or Methods section.

n/a	Confirmed	
☐	☒	The exact sample size (n) for each experimental group/condition, given as a discrete number and unit of measurement
☐	☒	A statement on whether measurements were taken from distinct samples or whether the same sample was measured repeatedly
☐	☒	The statistical test(s) used AND whether they are one- or two-sided<br>*Only common tests should be described solely by name; describe more complex techniques in the Methods section.*
☐	☒	A description of all covariates tested
☐	☒	A description of any assumptions or corrections, such as tests of normality and adjustment for multiple comparisons
☐	☒	A full description of the statistical parameters including central tendency (e.g. means) or other basic estimates (e.g. regression coefficient) AND variation (e.g. standard deviation) or associated estimates of uncertainty (e.g. confidence intervals)
☐	☒	For null hypothesis testing, the test statistic (e.g. F, t, r) with confidence intervals, effect sizes, degrees of freedom and P value noted<br>*Give P values as exact values whenever suitable.*
☒	☐	For Bayesian analysis, information on the choice of priors and Markov chain Monte Carlo settings
☒	☐	For hierarchical and complex designs, identification of the appropriate level for tests and full reporting of outcomes
☒	☐	Estimates of effect sizes (e.g. Cohen's d, Pearson's r), indicating how they were calculated

Our web collection on statistics for biologists contains articles on many of the points above.

Software and code

Policy information about availability of computer code

Data collection	No software was used for data collection
Data analysis	QuantaSoft Software, version 1.7, Regulatory Edition<br>FlowJo Software, version 10.9<br>LymphoTrack Software - MiSeq, version 2.4.3<br>Treemap Software, version 2019.9.1<br>Past4: Paleontological Statistics Software, version 4 by Øyvind Hammer<br>IMGT: ImMunoGeneTics Software, version 1

For manuscripts utilizing custom algorithms or software that are central to the research but not yet described in published literature, software must be made available to editors and reviewers. We strongly encourage code deposition in a community repository (e.g. GitHub). See the Nature Portfolio guidelines for submitting code & software for further information.

Data

Policy information about availability of data

All manuscripts must include a data availability statement. This statement should provide the following information, where applicable:

- Accession codes, unique identifiers, or web links for publicly available datasets
- A description of any restrictions on data availability
- For clinical datasets or third party data, please ensure that the statement adheres to our policy

The TRB and TRG sequencing data as well as the ITR-seq and ONT long-read sequencing data generated in this study have been deposited in the Sequence Read Archive (SRA) database under accession number: PRJNA926613 (https://www.ncbi.nlm.nih.gov/bioproject/PRJNA926613/). Source data are provided with this paper.

Research involving human participants, their data, or biological material

Policy information about studies with human participants or human data. See also policy information about sex, gender (identity/presentation), and sexual orientation and race, ethnicity and racism.

Reporting on sex and gender	The samples are provided anonymously and are stripped of any information (ID, birth term, gender, sex, genotype).
Reporting on race, ethnicity, or other socially relevant groupings	The samples are provided anonymously and are stripped of any information (ID, birth term, gender, sex, genotype).
Population characteristics	Randomized cord blood units that are otherwise discarded due to insufficient volume for public storage were used in the research. The samples are stripped of any information (ID, birth term, sex, genotype).
Recruitment	Human cord blood-derived CD34+ HSPCs, irrespective of sex, were obtained from Sheba Medical Center cord blood bank under Institutional Review Board-approved protocols (Approval 3500-16-SMC). Donations of cord blood are collected from the obstetric delivery department after informed consent is received allowing for cord blood units that are not suitable for banking to be used for research purposes. No compensation is given to the donors upon consent.
Ethics oversight	Sheba Medical Center Institutional Review Board (Approval 3500-16-SMC)

Note that full information on the approval of the study protocol must also be provided in the manuscript.

Field-specific reporting

Please select the one below that is the best fit for your research. If you are not sure, read the appropriate sections before making your selection.

☒ Life sciences ☐ Behavioural & social sciences ☐ Ecological, evolutionary & environmental sciences

For a reference copy of the document with all sections, see nature.com/documents/nr-reporting-summary-flat.pdf

Life sciences study design

All studies must disclose on these points even when the disclosure is negative.

Sample size	All data represents minimum N=3 and maximum N=14 biologically unique donors from CD34 HSPC cord blood samples. Exact p-value calculations are provided, using different test approached, adjusted to the sample size and configuration.
Data exclusions	There was no data exclusion in this study.
Replication	All experiments contain minimum N=3 independent biological experiments. All replications were successful.
Randomization	Each CD34 donor was used for all control ad test groups in a given replicate, thus elimination the need for randomization.
Blinding	Blinding was not necessary since all analyses were done in an unbiased way by machines and analytical softwares such as immunophenotyping by FlowJo and TCR analysis by NGS

Reporting for specific materials, systems and methods

We require information from authors about some types of materials, experimental systems and methods used in many studies. Here, indicate whether each material, system or method listed is relevant to your study. If you are not sure if a list item applies to your research, read the appropriate section before selecting a response.

Materials & experimental systems

n/a	Involved in the study
☐	☒ Antibodies
☒	☐ Eukaryotic cell lines
☒	☐ Palaeontology and archaeology
☒	☐ Animals and other organisms
☒	☐ Clinical data
☒	☐ Dual use research of concern
☒	☐ Plants

Methods

n/a	Involved in the study
☒	☐ ChIP-seq
☐	☒ Flow cytometry
☒	☐ MRI-based neuroimaging

Antibodies

Antibodies used

PE/Cy7-anti-CD7 1:20 (clone: CD7-6B7, BioLegend), BV421-anti-CD5 1:20 (clone: UCHT2, BioLegend), PE-anti-CD1a 1:5 (clone: BL6, Beckman Coulter), APC-anti-NGFR 1:20 (clone: ME20.4, BioLegend), PE/Cy7-anti-CD4 1:20 (clone: RPA-T4, BioLegend), APC-r700-anti-CD8a 1:20 (clone: RPA-T8, BD Horizon™) BV421-anti-CD3 1:20 (clone: UCHT1, BioLegend), and PE-anti-TCR PAN γ/δ 1:5 (clone: IMMU510, Beckman Coulter) antibodies.

Validation

PE/Cy7-anti-CD7 1:20 (clone: CD7-6B7, BioLegend) - https://www.biolegend.com/de-de/products/pe-cyanine7-anti-human-cd7-antibody-9938?GroupID=BLG10166
BV421-anti-CD5 1:20 (clone: UCHT2, BioLegend) - https://www.biolegend.com/en-gb/sean-tuckers-tests/brilliant-violet-421-anti-human-cd5-antibody-7308?GroupID=BLG5902
PE-anti-CD1a 1:5 (clone: BL6, Beckman Coulter) - https://www.beckman.pt/reagents/coulter-flow-cytometry/antibodies-and-kits/single-color-antibodies/cd1a/A07742
APC-anti-NGFR 1:20 (clone: ME20.4, BioLegend) - https://www.biolegend.com/fr-ch/cell-health/apc-anti-human-cd271-ngfr-antibody-6877
PE/Cy7-anti-CD4 1:20 (clone: RPA-T4, BioLegend) - https://www.biolegend.com/fr-fr/cell-health/pe-cyanine7-anti-human-cd4-antibody-829
APC-r700-anti-CD8a 1:20 (clone: RPA-T8, BD Horizon™) - https://www.bdbiosciences.com/en-us/products/reagents/flow-cytometry-reagents/research-reagents/single-color-antibodies-ruo/apc-r700-mouse-anti-human-cd8.565165
BV421-anti-CD3 1:20 (clone: UCHT1, BioLegend) - https://www.biolegend.com/en-us/products/brilliant-violet-421-anti-human-cd3-antibody-7153
PE-anti-TCR PAN γ/δ 1:5 (clone: IMMU510, Beckman Coulter) - https://www.beckman.co.il/reagents/coulter-flow-cytometry/antibodies-and-kits/single-color-antibodies/tcr-pan-g-d/im1571u

Flow Cytometry

Plots

Confirm that:

☒ The axis labels state the marker and fluorochrome used (e.g. CD4-FITC).

☒ The axis scales are clearly visible. Include numbers along axes only for bottom left plot of group (a 'group' is an analysis of identical markers).

☒ All plots are contour plots with outliers or pseudocolor plots.

☒ A numerical value for number of cells or percentage (with statistics) is provided.

Methodology

Sample preparation

CD34+ HSPCs were cultured in the StemSpan™ T-Cell Generation Kit (STEMCELL Technologies, Inc.). For the first 14 days, cells were cultured in StemSpan™ SFEM II medium containing Lymphoid Progenitor Expansion Supplement in plates pre-coated with the Lymphoid Differentiation Coating Material. Cells were then harvested and re-seeded for an additional 14 days in StemSpan™ SFEM II medium containing the T-Cell Progenitor Maturation Supplement on pre-coated plates. Flow cytometry analysis was conducted on days 14 and 28 of IVTD using the LSR Fortessa™ (BD Biosciences). On day 14 of IVTD, cells were stained with PE/Cy7-anti-CD7 1:20 (clone: CD7-6B7, BioLegend), BV421-anti-CD5 1:20 (clone: UCHT2, BioLegend), PE-anti-CD1a 1:5 (clone: BL6, Beckman Coulter), and APC-anti-NGFR 1:20 (clone: ME20.4, BioLegend) antibodies. On day 28 of IVTD, cells were stained with PE/Cy7-anti-CD4 1:20 (clone: RPA-T4, BioLegend), APC-r700-anti-CD8a 1:20 (clone: RPA-T8, BD Horizon™) BV421-anti-CD3 1:20 (clone: UCHT1, BioLegend), PE-anti-TCR PAN γ/δ 1:5 (clone: IMMU510, Beckman Coulter), and APC-anti-NGFR 1:20 (clone: ME20.4, BioLegend) antibodies. BD Horizon™ Fixable Viability Stain 510 was performed on all collected cells at both time points.

Instrument

LSR Fortessa (BD Biosciences)

Software

FlowJo Software

Cell population abundance

CD34 HSPCs were sorted and found to be 100% in the post-sort population.

Gating strategy

Gating strategies were based on fluorescence minus one (FMO) plus isotype control (at equivalent concentration to its antibody pair) samples using the following isotypes: PE/Cy7 Mouse IgG2a κ, (BioLegend), BV421 Mouse IgG1 κ (BioLegend), PE Mouse IgG1 κ (BioLegend), PE/Cy7 Mouse IgG1 κ (BioLegend), APC-r700 Mouse IgG1 κ (BD Biosciences), and APC Mouse IgG1 κ (BioLegend).

☒ Tick this box to confirm that a figure exemplifying the gating strategy is provided in the Supplementary Information.
