# Supplementary material for: CRISPR-Cas9 engineering of the RAG2 locus via complete coding sequence replacement for therapeutic applications
Source: Nat Commun. 2023 Oct 27;14:6771. doi: 10.1038/s41467-023-42036-5 (PMC10611791; doi:10.1038/s41467-023-42036-5)
Supplement: Supplementary file 3 — Description of Additional Supplementary Files [file 41467_2023_42036_MOESM3_ESM.pdf]

Title: Supplementary Data 1
Description: rAAV Donor Sequences – all of the exact sequences of the different donors

Title: Supplementary Data 2
Description: Representative ONT HDR reads – an example read of perfect HDR for both donors along with the reference expected sequence following perfect HDR for the two donors
